# Vocational Service Models and Approaches to Improve Job Tenure of People With Severe and Enduring Mental Illness: A Narrative Review

**DOI:** 10.3389/fpsyt.2021.668716

**Published:** 2021-07-09

**Authors:** Caitlin McDowell, Priscilla Ennals, Ellie Fossey

**Affiliations:** ^1^NorthWestern Mental Health, Melbourne, VIC, Australia; ^2^Centre for Youth Mental Health, University of Melbourne, Melbourne, VIC, Australia; ^3^Orygen, Melbourne, VIC, Australia; ^4^Neami National, Melbourne, VIC, Australia; ^5^Department of Occupational Therapy, Monash University, Melbourne, VIC, Australia; ^6^Living With Disabilities Research Centre, La Trobe University, Melbourne, VIC, Australia

**Keywords:** vocation, employment, job tenure, job retention, individual placement and support model, mental illness, psychiatric disability, psychosocial disability

## Abstract

Employment is a valued occupation that offers a sense of meaning, identity, and belonging. For people with severe and enduring mental illness, employment has also been associated with personal recovery and decreased use of mental health services. However, this population continues to be underrepresented in the labor market. Sustainable employment is often challenging for people with severe and enduring mental illness, due to a combination of personal, organizational and systemic issues. While Individual Placement and Support is an evidence-based model of employment support known to improve job attainment for people with mental illness, job retention and sustained workforce participation continue to be challenges. This narrative literature review was undertaken to address the question: “What vocational service models and approaches improve job tenure for this population?” CinAHL, Medline, Embase, PsycINFO, and Cochrane Library were searched for the period 2005–2020, using key terms and subject headings, including “severe mental illness,” “psychiatric disabilit^*^,” “job tenure,” and “job retention.” Several adjunct interventions may enhance job retention, including skills training, cognitive interventions, psychological interventions, and supported education, while social firms offer a different approach focused on creating new, sustainable job opportunities. Peer support and support from family and friends also appear to be important, and emerging evidence suggests that employment specialist practices, technology, self-management, and workplace accommodations may each also influence job tenure. Service providers could make more use of these non-clinical vocational approaches to improve employment retention for people with severe and enduring mental illness.

## Introduction

Employment is a social determinant of health and a right of citizenship, supported by *The United Nations Declaration of Human Rights, Article XXIII* and many national and state policies internationally (1). Employment offers financial stability and social inclusion, while unemployment and precarious employment are associated with poor health, financial stress, and social isolation (2). Employment is a valued social role and is considered an important part of recovery by many people with severe and enduring mental illness (3), yet this group continues to be underrepresented in the labor market (4, 5). Individual Placement and Support (IPS) is an evidence-based model of employment support for people with severe mental illness. IPS has clearly defined principles including competitive employment, zero exclusion, rapid job placement and integration between mental health and employment supports that are measured against a fidelity scale (6). Many studies have demonstrated that IPS achieves significantly better employment outcomes than other vocational programs, such as prevocational training and sheltered workshops (7). Nevertheless, IPS does not result in employment outcomes for many participants, and the positions obtained through this approach to employment support are often unskilled, precarious, and job tenure may be relatively brief (8). For example, average tenure in jobs obtained through supported employment has been reported as 18–30 weeks (9). In Australian Disability Employment Services, a lower job retention rate was found among people with psychiatric disabilities than any other disability group, including people with intellectual, physical, or sensory disabilities (10). Furthermore, the percentage of clients with psychiatric disabilities with high support needs who gained employment for at least 26 weeks was only 14.2% (10). Unsatisfactory job terminations have been commonly reported where a client is fired or quits without having other job plans (11, 12).

Maintaining employment may be particularly challenging for people with severe mental illness for a range of complex reasons, such as stigma and discrimination, lack of support in the workplace, or issues with managing fluctuating health conditions (13–15). A review of factors impacting job tenure for this population identified three main factors - worker's experience of the job, natural supports at work, and strategies for integrating work and wellness (9). According to a qualitative study of 51 people with mental illness, job tenure was most impacted by their interest in work, level of competency, and support from supervisors and coworkers (16).

There are differing ways to define and measure job tenure, resulting in inconsistent definitions across studies (9). Job tenure typically refers to the length of time spent in a job, rather than across multiple jobs (9). Most researchers have defined job tenure as the length of time in the longest held job during the study period, whereas others described job tenure as the length of time in any job or the duration of the longest held job. Furthermore, some studies only reported job tenure for competitive paid work or for job placements that were held for a minimum period (e.g., at least 5 days). Finally, different units of measurement were used across studies - usually days or weeks in a specific job, rather than employment across jobs. Variations in follow-up periods also confound study findings reporting the number of people employed or job tenure at the end of follow-up, with tenure likely to be underestimated where follow-up is brief. These inconsistencies hamper efforts to compare and generalize results of studies related to job tenure. Furthermore, Bond and Kukla (17) argued that cumulative time in employment may be a more meaningful measure of vocational success than job retention in a single job, as the goal is sustained employment over the long term. Limited research has explored the long-term employment trajectories of people with severe and enduring mental illness (18), whether employed via an IPS program or another approach.

Several reviews have explored the effectiveness of interventions that enable people with severe and enduring mental illness to obtain jobs (19, 20). However, there is less guidance for mental health service providers about what they can do to support people with severe mental illness to sustain employment. Therefore, the purpose of this review is to explore what vocational service models and approaches improve job tenure for this population. A narrative literature review of peer reviewed studies (21) was undertaken, guided by the question: “What helps to improve job tenure for people with severe and enduring mental illness?” CINAHL, Medline, Embase, PsycINFO, and Cochrane Library were searched for the period 2005–2020, using key terms and subject headings, including “severe mental illness,” “psychiatric disabilit^*^,” “job tenure,” and “job retention.” Citation scanning and reference checking of relevant articles was conducted. This review focuses specifically on identifying and organizing the available peer reviewed research about ways to improve sustainable employment for people with severe and enduring mental illness, so to guide service providers' practice in this area.

## Models of Supported Employment

Supported employment programs aim to assist people with mental illness or other disabilities to gain and maintain competitive employment. Individual Placement and Support (IPS) is the current best practice model of supported employment for people with severe mental illness (7). It includes eight core principles, including a focus on competitive employment, rapid job search, time unlimited support, attention to clients' interests, and integration of mental health and employment services (6). The terms IPS and supported employment are sometimes used interchangeably, but the key criteria that differentiate IPS from the broader definition of supported employment are that employment services are integrated with mental health services and that there are no exclusion criteria to access services. In high fidelity IPS programs, services should be accessible to anyone with mental illness who has a desire to work, and caseloads should be limited to 15–20 participants per employment specialist (22).

A systematic review and meta-analysis of international evidence found that IPS was more than twice as likely to lead to competitive employment than traditional vocational rehabilitation (20). Another recent meta-analysis of 30 IPS trials, predominantly in North America and other high income countries, also found that IPS participants had better vocational outcomes, including job tenure in longest held job and total job length of competitive employment across one or more jobs, compared to those who received treatment as usual (19). Similarly, a secondary analysis of data from four RCTs found that young people with severe mental illness (under 30 years old) experienced significantly longer job tenure with high fidelity IPS than alternative vocational service models (20 and 5 weeks, respectively; *p* < 0.001) (23). Furthermore, a recent RCT in the UK compared IPS to a time-limited version of IPS for people with mental illness, where job seeking support was limited to 9 months and post-placement support was limited to 4 months (24). The rationale for this was that the vast majority of job placements were attained within 9 months of IPS provision. Participants of time-unlimited IPS had marginally longer job tenure than those in time-limited IPS (median duration of longest employment of 124 days compared to 119.5 days), but this difference was not significant.

In summary, the best available evidence suggests that IPS programs achieve superior employment outcomes compared to alternative vocational services (7, 19). However, approximately half of IPS participants do not achieve a job placement, and job tenure remains brief - often <6 months for those who gain employment (9). Less is understood about the impact of short-term job tenure and how this may be impacting long-term employment outcomes for people with mental illness, however job loss is known to erode self-confidence and reduce optimism for future employment in the general population (25). To address these issues, researchers have investigated augmenting IPS with other interventions, such as job-related skills training or symptom-related skills training (e.g., cognitive training or social skills training). A recent Cochrane review analyzed data from 13 RCTs of supported employment augmented with another intervention (7). Participants receiving augmented supported employment worked more weeks than participants of alternative employment interventions, including prevocational training and transitional employment. Augmented supported employment was also more effective for maintaining competitive employment than supported employment alone, although this difference was small (7). Given the modest improvements in employment outcomes and low to moderate quality of studies to date, further research is required to determine effectiveness, as well as cost-effectiveness, of delivering IPS alongside adjunct interventions (7). The adjunct interventions that appeared most promising in this Cochrane review were symptom-related skills training programs focused on social skills or cognitive remediation.

## Adjunct Interventions

Several studies and literature reviews suggest that augmenting supported employment with other interventions may improve employment outcomes, including job tenure (7, 26–28). The following section groups, describes, and reviews approaches identified in the literature that enhance job tenure for people with severe and enduring mental illness. These approaches are summarized in Table 1, with brief rationales as to how each approach impacts job tenure for this population.

**Table 1:** A summary of approaches used to enhance job tenure.

**Approaches to enhance tenure**		**Rationale for how approach enhances job tenure**	**Key features of approach and strategies used**	**References**
Skills training	Workplace fundamentals: group skills training approach focused on general skills for work	Improvement in generic work skills is likely to enhance job tenure	Manualized modules addressing; problem-solving, managing stress, socializing at work & improving performance. Role play, individualized planning, homework	(29–32)
	Social skills training	Tailored work-related social skills training to address social skill challenges experienced by people with schizophrenia in sustaining employment (33)	Work related social skills training (33)	(34, 35)
Cognitive interventions	Cognitive remediation	Cognitive remediation (systematic efforts to learn and practice more effective cognitive strategies) addresses cognitive impairment that hampers job retention for people with schizophrenia	Computer-based cognitive training with/or without individual/group instruction such as Neurocognitive Enhancement Therapy (NET)	(36–38)
			Thinking Skills for Work – 7–11month program of cognitive training through computer based cognitive exercises and coaching from a Cognitive Specialist to learn and practice cognitive self-management strategies	(39–41)
	Cognitive remediation + employment support and social skills training	Addressing both cognitive impairment and social skills deficits will enhance job tenure	6 h pw computer training and 10 session social skills training	(42)
	Errorless learning	Acquisition of new work-related skills while avoiding reinforcement of errors in learning likely to enhance job tenure	Verbal/visual cuing, backward or forward chaining to support learning	(43, 44)
Psychological interventions	Cognitive behavioral interventions	Targeting maladaptive beliefs that are barriers to maintaining job likely to enhance job tenure	For example, CBT for Work Success: 12 × 1 h sessions on work goals, self-efficacy, motivation and coping strategies	(45–49)
	Motivational interviewing	Addressing motivational issues may improve capacity to sustain employment	A counseling approach used to facilitate change and attain goals	(50–54)
Supported education	Support (based on IPS principles) to enroll in and complete qualification provided when education/ qualifications identified as goal	Increased education/qualifications enable greater job choice & skills for work. Increased fit and satisfaction with employment likely to enhance job tenure	Support to engage in mainstream education based on IPS principles	(55, 56)
Social firms	Businesses employing a significant proportion of employees with disability/disadvantage and pay at market rates	The supportive and flexible work environments of social firms (enhanced supports and accommodations and decreased stigma) are likely to enhance tenure	Workplace accommodations and natural supports likely to facilitate the acceptance, satisfaction and integration of workers are freely available and enhance worker-job fit	(57–61)
Peer support	Additional support from peers to anticipate and problem solve issues at work	Unique lived experience and attitude of peer worker enables support and encouragement toward sustaining employment not available from other workers	Peer workers trained in IPS provided IPS interventions	(62)
Support from family and friends	Encouragement, problem solving, practical support	Timely supports from family and friends to provide encouragement or address challenges that arise at work likely to enhance tenure	Supports included friendship, understanding, inspiration, encouragement, financial support, transport, support to build routine	(63, 64)
Employment specialist practices	Recovery focused principles/ client-centered approach/ strong working alliance (beyond IPS)	Strong therapeutic alliance between employment specialist and employee leads to improved results including enhanced tenure	Relationship felt like a friendship and employee felt valued and hopeful about employment	(49, 65)
	Building and maintaining relationships with employers	Support provided to employers in the context of an existing strong relationship, when problems arise for an employee, are likely to enhance job tenure	Building and maintaining relationships with employers and working collaboratively with them to address any difficulties that arise	(66)
	Strong emphasis on job matching	Strong person-job fit likely to enhance job tenure	Attention paid to skills and preferences of the job seeker including how these change over time once employed	(67–69)
Technology	Technology based tools to support recovery, engagement and collaboration with support providers or workflow.	Technology based solutions that can be tailored to challenges faced by individuals in the workplace or in sustaining employment are likely to enhance tenure	Working well mobile app to support task management, stress management, and support from family, peers and professionals	(70)
Self-management	Health Optimisation for Employment (HOPE)	Self-management skills and strategies may improve job tenure	Structured, 20 h self-management program for job seekers with mental illness; cofacilitated by a peer educator	(71)
	Wellness Recovery Action Plan (WRAP)	Enhanced hopefulness, self-advocacy and symptom management influenced by WRAP (understanding of self, health and wellness, and skills and strategies to support self-management) are likely to enhance job tenure	WRAP tools learnt through 8 week WRAP course can be used to support job retention by managing daily stressors, using supports, taking responsibility, managing difficult situations and accepting feedback	(72)
Workplace accommodations	Negotiation of accommodations – reliant on disclosure of MI	Increased use of multiple accommodations is likely to increase tenure	Awareness of, and uptake of, the range of accommodations available under legislation such as flexibility, additional training or supervision, ability to swap tasks with colleagues, or recognition from employer	(73, 74)
	Disclosure of MI	Disclosure of MI to employer increases access to accommodations and understanding from employer that may enhance job tenure	Employment specialists can support development of disclosure plans – what to disclose and to whom – that weigh benefits (access to accommodations) and costs (stigma, discrimination)	(75–77)

### Skills Training

Skills training has been proposed as an intervention to improve job tenure of supported employment clients (29). The standardized “workplace fundamentals” program is an example of a skills training program developed for people with severe mental illness by Wallace et al. (78). It consists of several manualized modules addressing workplace skills, such as problem-solving, socializing with coworkers, managing stress, and improving performance, designed to develop skills applicable for work in general. The program involves role playing, using video-taped scenarios, developing and evaluating individualized plans to resolve workplace issues and homework assignments. It is held weekly for 6 months, then booster sessions run fortnightly then monthly up to 12 months (30). Proponents of IPS caution against delaying job searching by providing prevocational training to unemployed clients, as the evidence suggests that such delays are related to poorer vocational outcomes (29), so most studies randomly allocated participants to the skills training group or control group after job placement. One small RCT found that this training program improved knowledge of workplace fundamentals, but there were no significant improvements in vocational outcomes such as job tenure or wages earned (29). Surprisingly, both the intervention and control groups had substantially longer job tenure (331.6 and 288.5 days) than the average job tenure reported in previous supported employment studies (114.7 days), which the authors suggested may have been attributed to the participants' relatively high level of education. Another similar RCT found the intervention group experienced less job turnover and greater job satisfaction, despite no significant differences in job attainment, hours worked, or wages earned (31). While the studies by Wallace et al. (31, 78) were conducted over 15 years ago, a more recent RCT investigating IPS plus the workplace fundamentals training found that participants did not have better job tenure or other employment outcomes compared to participants of IPS alone (32). In comparison, another recent RCT (30) combining IPS with Workplace Fundamentals training and supported education for people with first episode psychosis attained better outcomes. The intervention group were employed, on average, significantly more weeks compared to the control group, who received equally intensive clinical care and conventional vocational rehabilitation (19 vs. 11 weeks of the 78 week study period; *p* = 0.04) (30). Job tenure also tended to be longer in the intervention group, although this difference was not statistically significant. Overall, there is currently limited evidence to suggest IPS plus generic workplace skills training improves job tenure and there is a lack of research conducted outside of North America.

Another type of skills training program was developed by Tsang and Mitchell (79) to improve social skills and social competence amongst people with severe mental illness, with an aim to gain and maintain employment. One study in Hong Kong found that this program did indeed improve job attainment and retention rates, and that follow up support further enhanced these outcomes (33). A further case study (80) and RCT in Hong Kong contribute to the evidence that social skills training may improve vocational outcomes for people with severe mental illness (34, 35). More specifically, participants in the IPS plus social skills training program were more likely to gain employment and have longer job tenure (46.9 vs. 36.2 weeks). In summary, social skills training seems a promising adjunct to supported employment services for this population.

### Cognitive Interventions

#### Cognitive Remediation

Cognitive impairment can be experienced by people with severe mental illnesses and is a predictor of psychosocial functioning and performance at work (81). A recent study investigating modifiable predictors of supported employment outcomes found that cognitive impairment was associated with shorter job tenure in competitive employment (82). Cognitive remediation is proposed as an intervention to improve vocational outcomes. Cognitive remediation programs often consist of computer-based cognitive training combined with individual and/or group instruction (83). One RCT found that cognitive remediation improved overall cognition and number of weeks worked in a sheltered work environment within an American psychiatric centre in the 12 month follow up period for people with enduring mental illness, predominantly schizophrenia, compared to the control condition (84). Another two American RCTs investigated the effectiveness of a cognitive remediation program called Neurocognitive Enhancement Therapy (NET) in combination with employment services for people with schizophrenia or schizoaffective disorders (37, 83). The first study with 145 veterans found that participants receiving NET and transitional sheltered employment worked significantly more hours (*p* < 0.03) during the 6 month follow up period than participants receiving transitional sheltered employment alone (37). Similarly, the second study found that participants receiving the combined interventions of NET and employment services (a hybrid transitional employment and supported employment program) attained better vocational outcomes, including job retention, compared to participants receiving only employment services (83). In contrast, a Japanese RCT did not find any difference in employment duration or other competitive employment outcomes, but the group receiving both cognitive remediation and supported employment experienced better social and cognitive functioning and reduced psychiatric symptoms (38). The authors suggested that perhaps with a longer follow up period, more significant employment outcomes would emerge.

The cognitive remediation program, Thinking Skills for Work, has also been associated with improved work outcomes when combined with supported employment services, including hours worked and wages earned, based on three RCT studies (39–41). One of these trials found the intervention group worked significantly more weeks in competitive employment (*p* < 0.001) (40), and another found the intervention group worked more weeks in their internship program but no difference in weeks worked in competitive employment (39). Finally, a larger RCT with 107 people who had been unsuccessful in high fidelity IPS-type supported employment found that those in the cognitive remediation and supported employment group achieved more job placements, weeks worked, and wages earned compared to the control group (41). Of the 52 people who gained employment in this study, participants in Thinking Skills for Work were more likely to have had successful jobs, a more successful work trajectory, and be employed at the end of the 2 year study period (85).

A further Chinese study investigated whether cognitive remediation improves outcomes for people with schizophrenia, when offered as an adjunct to supported employment and social skills training (42). The intervention group received 6 h per week of individualized, computer-based cognitive exercises and the control group attended sessions of television watching for 12 weeks prior to commencing supported employment. Both groups also completed a 10 session work-related social skills training program. Both the intervention group and control group improved over time, however there were no significant differences between groups on employment, clinical, or cognitive outcomes (42). Some other studies have indicated that cognitive remediation may be useful, but have not necessarily investigated its impact on job tenure (86, 87).

The participants, program components, and mode of delivery varied in each of these studies. While modest gains in cognition and vocational functioning have been reported across most studies, research to date has not consistently focused on sustained competitive employment as an outcome. More research is needed to determine which aspects of cognitive remediation programs are effective and for whom (88). Cognitive remediation programs may also be challenging for most service providers to deliver, given current qualifications, skills, and resources. However, there may be specific strategies that are feasible and effective to implement. Survey research by McGurk and Mueser (89) found that employment specialists used a range of strategies to help clients with severe mental illness to compensate for cognitive impairments across four domains: problem solving, memory, attention, and psychomotor speed. Employment specialists who coached clients to use a larger number of cognitive strategies tended to have a higher proportion of their caseload in employment (89).

#### Errorless Learning

Errorless learning is a learning strategy designed to assist people with cognitive impairment to acquire new skills. In contrast to learning through trial-and-error, an individual learns something by doing it, while not being given the opportunity to make mistakes. In the context of employment, the employment specialist or job coach provides physical or verbal cues to facilitate successful performance of new skills. The theory is that erroneous actions are not reinforced through implicit memory. Techniques may include providing cues verbally or through images, and backward chaining or forward chaining to gradually master all of the steps of a sequence. Some researchers have proposed that errorless learning may be suitable for people with schizophrenia, as it is common to have issues with their explicit memory and related cognitive systems, while their implicit or procedural memory may remain relatively effective (90). One study found that errorless learning was indeed effective for improving accuracy of work performance in entry level job tasks, however speed was not improved (90).

More recently, an RCT investigating errorless learning plus IPS was conducted with 76 veterans with schizophrenia or schizoaffective disorder (43). There were no significant differences between the intervention and control groups in the number of weeks worked. Only a small proportion of participants gained employment during the study (26% of participants gained competitive employment and 10.5% gained transitional work). Fifty percent of these quit their jobs within 10 weeks (43). When the results of the Kern et al. (43) study were combined with a study of participants of a community mental health center, the IPS plus errorless learning group maintained employment for significantly longer than those in IPS alone (32.8 vs. 25.6 weeks; *p* = 0.044) (44). While the evidence supporting errorless learning in vocational rehabilitation is limited, it appears to have potential to improve work performance and tenure for supported employment participants with severe mental illness, particularly those with cognitive impairment who are placed in entry level positions.

### Psychological Interventions

#### Cognitive Behavioral Interventions

Cognitive behavioral interventions have also been proposed to improve employment outcomes by targeting maladaptive beliefs related to employment that may be a barrier to gaining and maintaining a job (45).

Preliminary results of a Canadian RCT found participants of an eight session, group-based CBT program plus supported employment (CBT-SE) worked slightly more consecutive weeks than participants of supported employment alone, although this difference was not significant (22.5 vs. 18.3 weeks) (45). The final results of this trial indicated that CBT-SE was associated with improvements in job attainment and hours worked per week; however, no increase in the number of weeks worked (46). Another RCT found that outpatients with schizophrenia or schizoaffective disorder receiving group-based and individual cognitive behavioral interventions worked significantly more weeks in a job placement than the control group matched for treatment intensity (*p* < 0.05) (47). This program was later adapted for supported employment participants with severe mental illness (91). The CBT for Work Success program includes 12 1-h sessions and “focuses on examining and modifying maladaptive thoughts related to work, enhancing work-related self-efficacy, improving motivation to work, identifying work goals and personal values tied to vocation, and increasing healthy coping strategies that can be applied across employment settings” (91). Twenty-four supported employment participants were recruited from a Veterans Affairs medical centre in the United States. Although there was no control group in this study, a slightly larger proportion attained employment than in their usual supported employment program alone (43 vs. 33%). Also, participants perceived that the CBT for Work Success program supported them to achieve their work goals (48). Given participants in this study were described as “non-responders” to supported employment alone since they had not gained employment after more than 12 months in the program, this suggests the program may be beneficial to augment supported employment, especially for those facing more barriers to securing employment. Further evidence is needed to clarify whether this approach improves job tenure and other employment outcomes.

#### Motivational Interviewing

Motivational interviewing is a counseling approach used to facilitate change and goal attainment (92). It has been suggested as an approach to address motivational issues and employment ambivalence amongst people with mental illness and their clinicians (50–53). A cluster randomized trial found that training clinicians in motivational interviewing was associated with increased placements in employment for IPS participants with early psychosis (50). Another study of 26 consumers with severe and enduring mental illness found that those who received motivational interviewing were significantly more likely to be in paid employment at the 12 month follow up than the control group (*p* = 0.03) (54). However, these findings are limited by the small sample size and lack of randomization. Overall, there is currently limited evidence to demonstrate that motivational interviewing improves job tenure, but it has potential as a feasible, low cost means to augment IPS.

### Supported Education

Supported education has been proposed as an enhancement to IPS to address its shortcomings, including brief job tenure (55). Supported education, drawing on principles of IPS, involves supporting people to access and successfully participate in post-secondary education. It is argued that higher educational attainment is possible with appropriate support and will enable people to become more marketable to gain desirable and higher skilled positions in increasingly competitive job markets (55). Supported education is receiving more attention in youth focused studies because post-secondary qualifications are seen as an underpinning for many career choices and because many similar aged youth are involved in post-secondary study. There have been some studies combining supported education and supported employment (56, 93), yet determining how the education component leads to improved job tenure and career development will require longer term studies that consider course completion, job success, and sustainability over time.

## Social Firms

Businesses that have a social purpose to create employment opportunities for people with mental illness or other disadvantage in the labor market are known internationally as social enterprises, social firms, or affirmative businesses. These businesses offer employment to an integrated workforce, including people with and without a known disability or disadvantage in the labor market. A review of 21 social enterprises in Ontario, Canada described them as “enabling spaces” that often offered more flexibility, security and on-the-job support than mainstream employers (94). A survey of social firms in the UK found that most employees with mental illness had been employed more than 2 years (57). Another cross-sectional study of Canadian social firms reported that the average length of job in social enterprises in Quebec was 77.8 months and in social enterprises in Ontario was 66.5 months, both of which are substantially longer than the average of 8 months reported in IPS trials (58). Finally, a recent scoping review of social firms research reported that average job tenure of people with disabilities ranged from 15 months (median) to 7.3 years (mean), with seven studies reporting an average job tenure of over 5 years (59). While these studies were not able to confirm causation of better job tenure, it is suggested that the availability of workplace accommodations without specific disclosure is associated with job retention.

Factors contributing to sustained employment and satisfaction with employment among employees with mental illness in a small Australian study at a social firm included the rewards, work schedule, task demands, and interactions with others in the workplace (60). Villotti et al. (61) found that training possibilities and schedule flexibility, in particular, were associated with increased job tenure in their cross-sectional study comparing social firms in Australia, Canada, and Italy (61). Gilbert et al. (57) argue that social firms can complement IPS services, and may be particularly beneficial for people who do not obtain employment through IPS or prefer an alternative to IPS, as well as being useful to increase the range of job options for disadvantaged workers in restricted labor markets. While the relatively better job tenure in social firms is generally considered a positive, some also argue that social firm employees may find it difficult to break into other forms of employment and hence these settings do not offer true social inclusion and citizenship (95).

## Other Supports for Sustaining Employment

### Peer Support/Social Support

Peer support may be helpful for dealing with work issues and maintaining employment, although the research is limited (68). Qualitative studies have highlighted the importance of peer and community supports for employees with severe mental illness, including friends, and peers from vocational groups, mental health groups, or spiritual groups (63).

A pilot evaluation of IPS delivered by peer employment specialists found 33% of participants got competitive jobs and the mean job tenure was 26.1 weeks (62). This study demonstrated that peer workers with no prior experience providing vocational services can be trained to deliver IPS with a fair level of fidelity. Their lived experience of maintaining employment while managing a mental illness may have enhanced their credibility with participants. While the employment outcomes were modest, the authors noted that when interpreting the employment outcome results, it's important to take into consideration the level of IPS fidelity, high unemployment levels during a recession and participant characteristics (e.g., Predominantly people with schizophrenia, ethnic minorities, and many had education as a primary goal rather than employment) (62).

Several studies reviewed by Williams et al. (9) highlight the importance of support from family, friends, clinicians and other support people to sustain ongoing employment. Qualitative studies in particular have identified support from significant others as important, in addition to support provided by employment specialists. For example, a study by Auerbach and Richardson (64) explored the long-term work experiences of six people with severe mental illness. This study found that personal supports such as family and friends, as well as therapists and employers, were important to help them process and solve problems in order to maintain employment. Another small study by Kennedy-Jones et al. (96) found that support from significant others was a key theme contributing to a sense of self as a “worker” for people with a severe and enduring mental illness. A larger study exploring how 32 people with psychiatric disabilities found and retained work also highlighted the importance of social support from family members to encourage and instill the belief that they could successfully maintain employment (63).

### Employment Specialist Practices

Employment specialist practices, such as establishing helpful relationships with employers and clients, likely affect job tenure for their clients with mental illness. Employment specialist practices and performance vary significantly within and between supported employment programs (97). Certain employment specialist characteristics and competencies appear to facilitate employment outcomes for clients with mental illness (66). Australian research described how recovery focused principles (98) supported the relational and strengths-based practice of employment specialists, arguing this contributed to positive employment outcomes, including sustained employment (65). Employment specialists' competency to build and maintain relationships with employers was significantly correlated with job acquisition and job retention (66). In addition, support and a client-centered approach was predictive of successful employment outcomes for clients with mental illness. The percentage of time employment specialists spent in the community and frequency of contacts with clients were positively correlated with employment outcomes in an IPS service for people with severe mental illness, including the proportion of their clients who had worked at least 90 consecutive days (99). Supervisor ratings of employment specialists' performance and efficacy were also predictive of employment outcomes, whereas self-reports were not (99).

Helpful relationships between professionals and clients are central to service delivery for people with severe and enduring mental illness. Also known as a working alliance or therapeutic relationship, a helpful relationship is one where clients “spend time with professionals that they know and trust, who give them access to resources, support, collaboration and valued interpersonal processes, which are allowed to transgress the boundaries of the professional relationship” (100). Helpful relationships in the context of IPS were related to providing responsive and practical professional support, treating clients as human beings, and doing “little extras” beyond what was expected (101).

A qualitative study by Gowdy et al. (67) investigated the differences between high performing and low performing supported employment programs in the United States. One of the key themes focused on the factors that related to helping clients maintain a job. Clients at high performing sites also felt they received a higher level of post-placement support from their employment specialists, for example, through on-site job coaching or negotiating adjustments in the workplace. Employment specialists at these sites appeared to develop stronger relationships with clients and put more emphasis on job matching to clients' interests (102). Another more recent qualitative study in Australia compared practices of high performing and low performing employment specialists (49). Performance was based on their rates of job attainment and retention for their clients with psychiatric disabilities. This article focused on engagement and support practices in particular. Some common practices used by high performing employment specialists included developing working alliances; addressing negative experiences (e.g., addressing previous work challenges, perceived barriers); using psychological interventions (basic psychological treatment techniques e.g., based on CBT, motivational interviewing, social skills training, problem solving); and supporting employers (e.g., educating employers about mental illness, sharing success stories) (49).

A literature review by Kirsh (68) also suggests that appropriate job matching is important for supporting job tenure. Another study examined the hypothesis that matching job seekers with mental illness to their preferred occupation improves job tenure and other employment outcomes (69). Over the 2 year study period, 147 of the 187 participants gained paid employment. More than half of those were at least roughly matched to one of their preferred jobs. Surprisingly, there was no significant correlation between job match and job tenure or other outcomes such as job satisfaction or hours worked (69).

### Technology

Technology also has the potential to enhance outcomes of supported employment services. The rise in accessibility of mobile technologies and internet provides an opportunity to offer technology-based interventions (e.g., tools to support recovery, education, and collaboration between support providers) and use technology to facilitate workflow (e.g., screening and assessment) (103). It can provide an additional mode of communication and engagement with disengaged or hard-to-reach populations and support fidelity of evidence-based programs (103). There is growing evidence of the effectiveness of internet-based interventions for people with severe mental illness and their clinicians, however there is currently very limited evidence regarding the use of technology to enable employment outcomes in particular. One small study focused on the development of the WorkingWell mobile app, designed to improve job tenure for people with severe mental illness (70). The majority of participants were comfortable using technology, owned mobile phones, and identified potential benefits of the app for supporting ongoing employment, such as managing tasks, managing stress on the job, and readily accessing support from professionals, family, and peers. Further innovation and research is warranted regarding the use of technology to help people with mental illness to gain and sustain employment.

### Self-Management

Self-management is key to sustaining employment according to qualitative research reporting mental health consumers' views (14). Therefore, structured self management and psychoeducation programs may be useful to augment supported employment. For example, the Health Optimisation Program for Employment (HOPE) was associated with improved self-efficacy and two-thirds of participants acquired a valued social role in the 6 months post-program in paid employment, volunteer work, or education (71). A qualitative study investigated the impact of Wellness Recovery Action Plan (WRAP) on employment for 10 people with mental illness, and suggests it facilitated employment, and that in turn, employment further supported recovery (72). Job tenure was not reported in this study, however an eligibility criteria to participate in the interview was that they worked at least 90 days after completing the WRAP training. There is a lack of RCTs and longer term studies to establish the effectiveness of self management programs for improving job tenure.

### Workplace Accommodations

Another factor that may affect job tenure for people with mental illness is the ability to negotiate reasonable accommodations in the workplace (74). Disability discrimination legislation entitles people with mental illness to reasonable workplace accommodations or adjustments in many countries, however people with mental illness are often unfamiliar of this legislation and how it applies to them (104). Many employment specialists and employers also have limited knowledge regarding the rights of people with mental illness to reasonable adjustments such as flexibility, additional training, or supervision (105). In some countries, such as Australia, government funding is available for reasonable workplace adjustments, however this is rarely accessed for people with mental illness (10). This is likely due to a range of factors including limited awareness among employment specialists, employers, and job seekers that this fund applies to people with mental illness. A scoping review regarding workplace adjustments for people with mental illness found that a larger number of workplace adjustments was associated with longer job tenure, although the evidence was limited (74).

Similarly, a study by Corbière et al. (73) found that availability of workplace accommodations and natural supports lowered the risk of people with severe mental illness losing their job. Supervisor and co-worker supports (informational, instrumental, and appraisal supports), and particularly rewards or recognition from the supervisor and the ability to exchange work tasks with colleagues were related to longer job tenure. People who disclosed mental illness to their employer had a significantly larger number of workplace accommodations available (*p* < 0.01), such as support from a job coach in the workplace (73).

The impact of disclosure on job tenure is still unclear and appears to be variable in different circumstances. Disclosure of personal health information in the workplace involves a complex decision-making process, as it is necessary to consider how much is appropriate to disclose and to whom (106). Disclosure of mental ill health was a negative experience for some participants in a qualitative study by Gladman and Waghorn (107). A study of 55 employed people with severe mental illness did not find any correlation between disclosure of mental illness to supervisors or co-workers and outcomes including job tenure or job satisfaction (75). In contrast, another small study of IPS participants found that those who disclosed their mental illness to a prospective employer were more likely to obtain jobs that matched their interests and have longer job tenure (32.5 vs. 12.5 weeks) (76). Seventy per cent of participants who disclosed obtained workplace adjustments. The most common was having contact with a job coach or other helping professional in the workplace, followed by having a modified work schedule (76). It appears that support is needed to optimize the benefits of disclosure (e.g., workplace supports and accommodations), while mitigating the risks, such as stigma and discrimination which can lead to distress and job termination (77). Employment specialists could support clients with mental illness to develop detailed plans for how they will manage their personal information in the workplace and negotiate reasonable workplace adjustments, with an aim to enhance outcomes including job tenure (77, 107).

## Discussion

This paper reviews the non-clinical interventions and approaches that service providers may implement to enhance sustainable employment outcomes for people with severe and enduring mental illness. Current thinking, practice and emergent evidence supporting a diverse range of interventions and approaches used to improve job tenure for this population is summarized. Systematic reviews have demonstrated that high-fidelity Individual Placement and Support (IPS) programs achieve better employment outcomes, in particular job attainment, compared to other supported employment models and traditional train-then-place employment programs (7, 19). Nevertheless, interventions to improve other employment outcomes such as job tenure are needed. Several adjunct interventions and approaches show promise for supporting job retention, including skills training, cognitive interventions, psychological interventions, and supported education, and warrant further research. Social firms offer an alternative approach by creating new employment options in the open, competitive labor market, which appear to be associated with longer job tenure. Social support including peer support and support from friends and family also appear to be important. Finally, there is emerging evidence to support the notion that employment specialist practices (e.g., job matching), technology, self-management, and workplace accommodations influence job tenure. The present state of evidence is variable or emergent for all the adjunct interventions discussed, so there is a clear need for further research.

The varied approaches to enhancing job tenure in this review identify a range of potential intersecting factors impacting tenure. They situate “the challenge” as predominantly within the person seeking employment or alternatively, within the contexts and structures of employment and society. Consequently, the interventions are designed to focus on job-seekers (e.g., cognitive remediation, psychological interventions, skills training), or on contexts, structures and supporters (e.g., social firms, peer support, family and friends, employment specialist practices, workplace accommodations). Interestingly, few studies attend to the role of local employment market and unemployment rates, or consider higher level structural reforms such as wage subsidies or employment/welfare policy. The available evidence does not provide clear guidance as to whether person or context focused interventions are more likely to deliver improved job tenure for people with severe and enduring mental illness. A recovery lens suggests that a single intervention is unlikely to meet the needs of diverse job seeking cohorts (108). While individual strategies need to be tested and compared in research trials it may be that a flexible and tailored approach, where an individual - their strengths, values, and challenges - can be known, and where interventions are selectively drawn on to meet these needs and contexts in a timely way. For example, developing cognitive strategies for people whose challenges include cognitive impairment, errorless learning for employees with memory problems, or social skills training for people experiencing interpersonal issues. In addition, availability of peer support could identify, normalize and enable timely problem solving around emerging challenges in the workplace, and potentially provide role models for people with severe mental illness. Families and significant others should also be better supported to instill hope, support problem-solving, and provide encouragement and practical support to enable job retention.

The role of employment specialists and the structure and timing of support provision requires further consideration to identify what works to improve job tenure and for whom. Drake et al. (97) asserted that “further work is clearly needed to clarify the critical skills of employment specialists, to develop ways to assess these skills, and to improve the skills through training and supervision” (p. 317), however focused attention must go to the critical points where employment becomes tenuous and the role specialists might play at these points. The role of training and support for employment specialists has been highlighted (62, 92) and there is also need for systems to support sharing of learning across vocational service providers so practices that support people to navigate moments of tenuous employment can be rapidly disseminated.

Most studies in this review included broad samples of participants with severe and enduring mental illness, without considering in a more focused way how challenges, needs and contexts might be differentiated. The ways in which systems are organized tends to group participants into defined populations, for example young people, or veterans, and may gloss over rather than reveal the range of employment trajectories, needs or challenges, and the supports best suited to individuals and sub-groups within this population. Further research needs to give more detailed consideration to the types of challenges that arise for people as they navigate employment in an effort to secure meaningful and sustainable employment and to pursue their career aspirations. Context is also critical, so additional attention needs to go to describing study contexts, including employment markets, support structures (including mental health services), and welfare policies that may play a role in outcomes. Qualitative studies may generate a clearer understanding of the range of challenges experienced by individuals, the contextual influences of welfare policies and employment markets, and the impact of gaining and then losing employment. Further research should focus on high quality randomized controlled trials of interventions, including more complex, clustering and tailoring of interventions matched to need, that may augment IPS. Alternatives to IPS are also needed for people who want to work but who do not benefit from IPS plus adjunct interventions. Longitudinal studies that could reveal longer term impacts and employment trajectories of people with severe and enduring mental illness are required. Given the research reviewed in this paper has been conducted predominantly in high income countries, there is also a need explore what promotes sustainable employment for people with mental illness in low and middle-income countries (7).

This narrative literature review provides an overview of peer reviewed literature focused on the broad range of interventions and approaches with potential to improve job tenure for people with severe and enduring mental illness. While a literature search was conducted, the quality of included studies was not assessed, meaning that this review may be subject to a greater degree of bias than a systematic review (21).

## Conclusion

Sustainable employment is an important social determinant of health and can offer financial stability, opportunities to participate and contribute, and facilitate social inclusion. On the other hand, unsatisfactory job conditions and job terminations may have negative impacts on mental health and sense of self-efficacy to pursue vocational goals in the future. Job retention is also a key concern for governments from an economic perspective (109). This review identifies a range of potential strategies that may enhance job tenure for people with severe and enduring mental illness. Given the substantial body of evidence supporting IPS for people with severe and enduring mental illness, it is strongly recommended to advocate for funding and policies to support implementation of high quality IPS within adult mental health services. Further to this, there is a need to establish adjunct approaches to assist those who need additional support to be successful in sustaining employment. In places where the labor market is particularly challenging, it is also important to advocate for job creation schemes that have strong tenure records, such as the establishment of social firms. This review reveals job tenure as a complex concept for people with severe and enduring mental illness. Thus, narrowly focused approaches may be inadequate to deliver improvements in job tenure. It is likely that job tenure will be enhanced through a nuanced understanding of individuals' needs, matched with selected strategies provided by people with the right skills, attitudes and supports, with adequate intensity, over an adequate time period.

## Author Contributions

CM and PE equally contributed to establishing the scope of this review. CM wrote the first draft of the review. PE and EF critically reviewed the draft manuscript. All authors contributed to finalizing and approving the manuscript for submission.

## Conflict of Interest

The authors declare that the research was conducted in the absence of any commercial or financial relationships that could be construed as a potential conflict of interest.
